# “I Have No Hope”: The Experience of Mothers in Polygamous Families as Manifested in Drawings and Narratives

**DOI:** 10.3389/fpsyg.2020.608577

**Published:** 2020-12-07

**Authors:** Faten Gadban, Limor Goldner

**Affiliations:** The School of Creative Arts Therapies, The Emily Sagol Research Center, Faculty of Social Welfare and Health Sciences, University of Haifa, Haifa, Israel

**Keywords:** polygamous families, mothers, internal distress, drawings, rejection

## Abstract

Polygamy is associated with lower marital satisfaction and is known to involve sexual, physical, and emotional abuse on the part of the husband. Less is known about the experience of mothers in polygamous families. This study was designed to shed light on the experiences of women in polygamous families in a sample of 80 Israeli Arab mothers living in polygamous families who use social services, domestic violence agencies, and health centers. Mothers were asked to draw their experiences in their families and to provide narratives for the drawings. A phenomenological approach was used to analyze the drawings, and yielded five different pictorial phenomena: (1) pseudo-sweetness, (2) houses, (3) the absentee father and the estranged mother, (4) incorporation of graphic symbols and lettering that represented distress, and (5) growth and development. Most of the drawings were restricted and shallow, indicating a complex emotional state of despair and distress. The central feelings that emerged from the drawings were negative emotions of anger, sadness, loneliness, and powerlessness. While some women longed for romantic relationships with their husbands, others expressed the desire for revenge and justice. Dissociation and parentification, as central coping strategies, emerged from the drawings and the narratives. The findings are discussed theoretically and clinically.

## Introduction

Polygamy is defined as a marriage in which one spouse (man or woman) has several other spouses. The most common form of polygamy is when a man has more than one wife (Al-Krenawi, 2012). Polygamy was practiced in numerous cultures in the past but is now mainly found in the Middle East, North Africa, East Asia, but also in certain European and North American communities (Al-Krenawi et al., 2006). Although there are no official data on the extent of polygamy in Israel, it is well known that it exists, and that most polygamous families in Israel are Bedouin. Most families live mainly in the Negev (i.e., the southern part of Israel), although polygamy has also been documented in several villages in the northern part of Israel (Layish, 2017). The Sharia court (Muslim religious court) allows a plurality of wives, provided that the husband can provide for the economic and emotional needs of all his wives and children equally. However, Israeli law considers polygamy to be a criminal offense for which the sentence can range up to 5 years of imprisonment (section 176 of the Penal Code, 1977).

Most polygamous marriages in Israel involve one man married to two women, although there are some marriages with men married to three or four women (Layish, 2017). Polygamy is practiced by both young and old men and also among men with higher education (Al-Krenawi, 2012). In many cases, it is accompanied by lower marital satisfaction and involves sexual, physical, and emotional abuse by the husband (Elbedour et al., 2006), which has severe psychological and physiological consequences for the wives, including low self-esteem, low life satisfaction, loneliness, depression, somatization, phobia, anxiety, and paranoia (Al-Krenawi and Slonim-Nevo, 2008b; Al-Krenawi, 2012; Daoud et al., 2014), as well as impaired socioemotional and academic adjustment in the spouses’ children (Al-Krenawi and Lightman, 2000; Elbedour et al., 2002).

Studies conducted in different countries have suggested that family functioning in polygamous families tends to be more problematic and impaired than in monogamous families (Al-Krenawi and Slonim-Nevo, 2008a). This is primarily because it involves co-wife jealousy, rivalry, and an unequal distribution of household and emotional resources (Slonim-Nevo et al., 2008), and can produce hostility among co-wives and between the children of the different wives (Al-Krenawi, 1998). The findings tend to indicate that the negative intra-psychic consequences for the mother affect the quality of her parental involvement and caregiving, which is manifested in low maternal involvement and monitoring, emotional detachment, and high levels of hostility toward her offspring (Elbedour et al., 2002; Al-Krenawi and Slonim-Nevo, 2008a). Nevertheless, several studies have indicated that the ability of members of polygynous families to function well depends on family connectedness, avoidance of minor conflicts, and the possibility for open communication and emotional ties among family members (Al-Krenawi, 1998; Slonim-Nevo et al., 2008). This suggests that the conflict resolution strategies that are adopted, rather than the structure of the relationship itself (i.e., polygamy vs. monogamy) predicts problematic family functioning. To better understand women’s internal experiences of their place in a polygamous family, this study examined drawings as a projective art-based technique associated with narratives in a sample of women in polygamous marriages in Israel.

### The Use of Drawings and Narratives

Empirical and clinical work in art therapy and allied fields have shown that drawings enable the expression of hidden or repressed thoughts and feelings in a relatively fast and straightforward way (Woolford et al., 2015). For researchers as well as therapists, drawings can shed light on individuals’ internal worlds (Gantt and Anderson, 2009) and their perceptions of their families, parents, siblings, or spouses (Goldner and Scharf, 2012). Drawings are considered to tap into conscious and unconscious content in way that communicates feelings and ideas in a safe environment (Malchiodi, 1997; Lev-Wiesel and Shvero, 2003; Jacobs-Kayam et al., 2013). The assumption is that by using colors, shapes, and motifs, as reflected in visual content, the unconscious can be expressed (Goldner and Scharf, 2012). The covert and overt ideas, distressful feelings and thoughts, and concerns and worries captured in the drawings add a layer of meaning to the verbal content (Lev-Wiesel and Liraz, 2007; McInnes, 2019).

Since stressful events are often recalled in a fragmented, dissociated, and nonverbal way, expressing this suffering through implicit symbolic modes of communication such as drawing can provide individuals with access to dissociated self-states (Van der Kolk, 2002) and possibly overcome the dissociative defensive mechanism of self-censorship, thus allowing the trauma to emerge in pictorial form (Lev-Wiesel, 2005; Avrahami, 2006; Huss et al., 2012). Clinical and empirical evidence suggests that drawings can reveal individuals’ perceptions of their pain, illness, and disabilities (Broadbent et al., 2009).

Recently, drawing has been integrated into interpretative phenomenological analysis (IPA). Boden et al. (2019) suggested the use of drawing and narratives as a vehicle to help participants explore and communicate their life worlds and the relational context of their distress. They argued that drawings accompanied by narratives activate several senses simultaneously, which provide rich data that captures the quality of the art maker’s inner and relational experience and helps further communication. This is especially beneficial for less verbalized populations (Lyon, 1995). The current study focused on the ways women from polygamous families perceive their place in their families through drawings and narratives.

## Materials and Methods

### Participants and Procedure

The sample was comprised of 80 Israeli Arab women from both the southern and northern parts of Israel, all of whom were in polygamous marriages. The participants were recruited through flyers and by word of mouth via social services staff in various community sites (e.g., social services, domestic violence agencies, and health centers). To better explore the perceived family dynamics of these women, including parenting, the criterion for participation in the study was to have at least one child. The mean age of the participants was 40.89 (SD = 9.87). The participants had been married on average for 19.88 years (SD = 10.37). All the women were Muslim. The average number of children in each marriage was 5.75 (SD = 2.89). Almost half of the participants were the second wives in the marriage order (*n* = 39, 48.7%), slightly fewer were the first wives (*n* = 35, 43.8%), and only 7.5% were the third or fourth wives (*n* = 4). The women had 8.89 years of education on average (SD = 4.25), and three-quarters of the participants were unemployed (*n* = 60, 75%). The participants were asked to indicate whether they described themselves as having a high, medium, or low socio-economic status. Approximately two-thirds (*n* = 49, 61.3%) of the participants reported that their socio-economic status was either low or medium.

The study was conducted in accordance with APA ethical standards for the treatment of human subjects. Ethical approval from the university of Haifa Ethics Committee (approval # 1809/19) was obtained. In compliance with ethical standards for this community, women were not asked to sign informed consent forms, since polygamy is prohibited by law in Israel, and signing the form would be an acknowledgment of violating the law. Instead, the first researcher signed an affidavit stating that the purpose of the study was made clear to the women and that they had agreed to participate in the study. Upon agreement, face-to-face individual meetings were conducted in the homes of the participants in Arabic by the first author, during which time she administered the drawing task. Participants were assured of the confidentiality of their responses. Participants were not compensated for their participation.

### Measures

#### The Drawing Task

Participants were handed a blank sheet of A4 (21 cm × 27.9 cm) paper, a pencil, an eraser, and a set of 12 colored markers, and were requested to draw an image that reflected their experience within the family and to provide a short narrative related to the drawing. The drawings could portray their experiences abstractly or figuratively. The narratives were audiotaped, transcribed into Arabic, and later translated into English to enable comparison.

The drawings were analyzed by applying the principles of IPA for drawings (Huss et al., 2012; Thyme et al., 2013). First, the form and content of the drawing was examined carefully (Kato and Morita, 2009) in order to identify repetitive pictorial features composed of pictorial art such as lines, colors, shapes, images, textures, contrasts, massive areas, spaces, and the relationships between them (Huss et al., 2012; Thyme et al., 2013; Boden et al., 2019). Symbols and pictorial phenomena were labeled based on the Jungian approach which posits that any symbol or pictorial phenomenon encompasses meaning beyond the object itself or the particular drawing (Eisenbach et al., 2015). In addition, dictionaries of symbols from the field of projective art-based techniques were consulted to define each symbol. The data that emerged from the analysis were then classified into broad, global, and aggregate pictorial phenomena based on the organization, the aggregation of signs in the drawings, and the general impression. In the next step, interpretive psychological theories that assess mental functioning through compositional and stylistic elements were applied to generate meaning from these phenomena (Huss et al., 2012). In addition, in this study, an attempt was made to examine the extent to which the women’s verbal narratives supported, contradicted, or added more layers of information to the pictorial phenomena identified from the drawings.

The drawings and the narratives were analyzed by the two authors, who are both experienced creative art therapists with extensive training in the phenomenological analysis approach to art therapy. To ensure inter-coder reliability, we conducted the pictorial phenomenological analysis and examined the narratives separately. Then, we compared individual analyses, discussed disagreements, and looked for common ground as to the visual content and meaning. The intra-class correlation coefficients (*Kappa*) for the drawing phenomenon based on 25 (31%) of the drawings ranged from 0.82 to 0.87. In addition, the narratives were coded as either positive or negative. Negative narratives were defined as those revealing feelings of despair and internal distress, whereas positive narratives were defined as those that expressed positive resolution and hope, either by personal growth or through their children’s development. The intra-class correlation coefficients (*Kappa*) for the narrative analysis based on 25 (31%) of the narratives was 0.87.

## Results

The women were asked to depict the ways they experienced their place in the family. The analysis of the drawings yielded five different pictorial phenomena: (1) pseudo-sweetness, (2) houses, (3) the absentee father and the estranged mother, (4) incorporation of graphic symbols and lettering that represented distress, and (5) growth and development. Most of the narratives (*n* = 64, 80%) were negative; the remainder (*n* = 16, 20%) were positive.

### First Phenomenon: Pseudo-Sweetness

**FIGURE 1:**
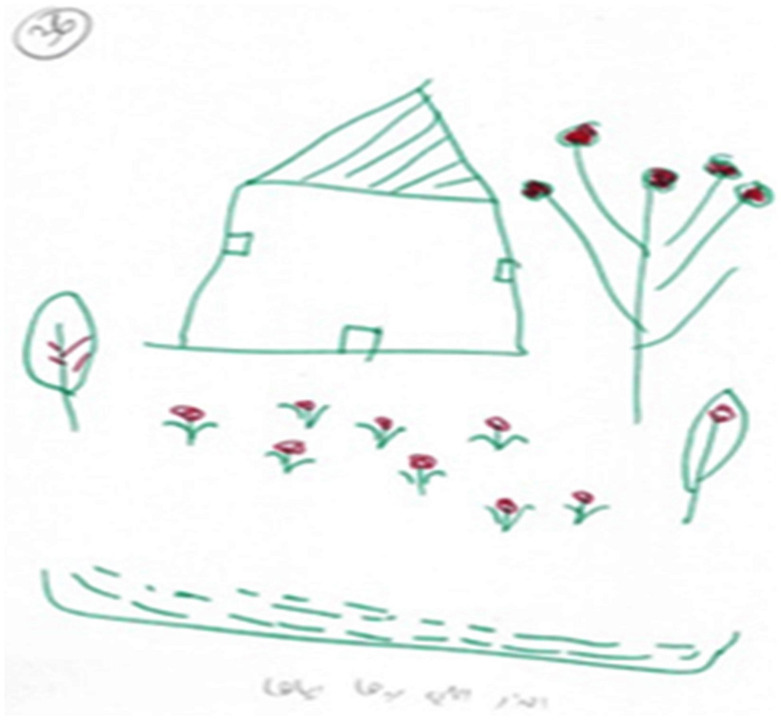
An example of a “Pseudo Sweet” drawing.

**FIGURE 2:**
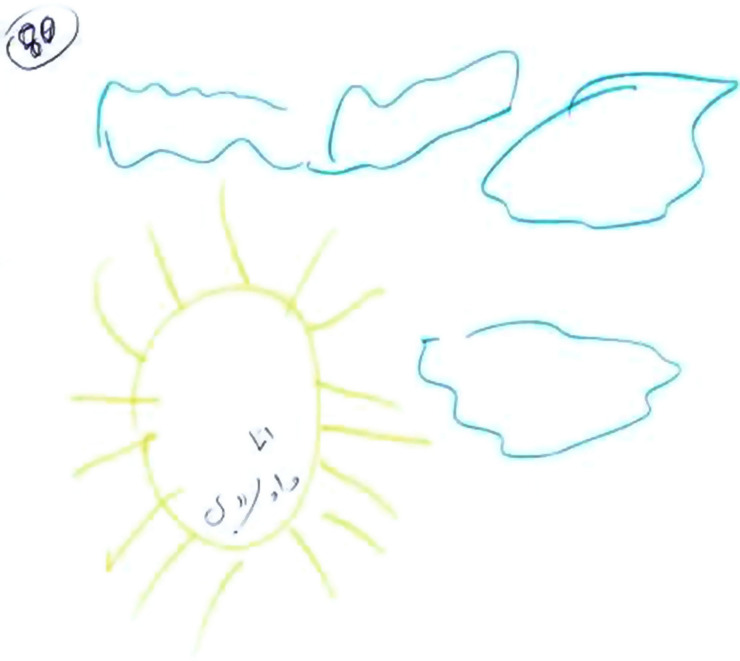
An Example of a “Pseudo Sweet” drawing.

The first phenomenon was termed pseudo-sweetness, and was present in more than a quarter of the drawings (*n* = 22). These drawings incorporated butterflies, birds, and hearts; however, further scrutiny indicated that most of these objects were moderately small, drawn on the margins of the page so that most of the page remained blank. Often, the drawings took on a childish quality. In addition, the color scale was relatively limited, the objects were disconnected and the family was not depicted (see Figures 1, 2). These all revealed the sense of impoverishment, sadness, anger, and pain.

Thus despite this attempt to sweeten reality and present it in a positive light, 14 of the narratives were characterized by hopelessness, despair, and helplessness, and suggested that they relied on their children as a source of comfort and strength. Eight mothers described how they implemented a defensive strategy of role reversal, which involved shifting from a negative to a positive description in what may have been an attempt to convince themselves that their reality was not so desperate. The narratives included statements such as “the situation in my home is tough, I have almost no contact with my husband. The most important thing is that I have my children”; “I don’t think that my bitter reality can be changed, this is my destiny, and I must accept it, this is God’s wish”; “I have no words, Al-Ahmadullah (thank God), my kids are healthy, and I don’t want any more than that.”

### Second Phenomenon: House Drawing

**FIGURE 3:**
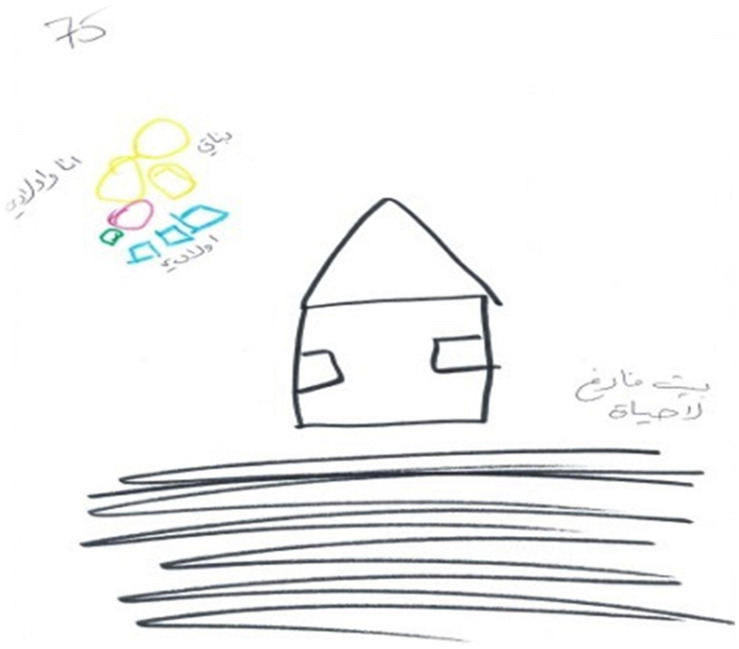
An example of a “House Drawing.”

**FIGURE 4:**
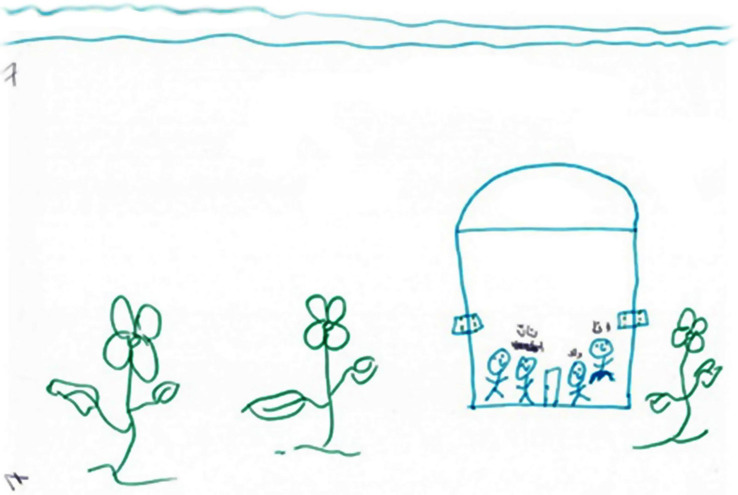
An example of a “House Drawing.”

The second phenomenon, which was evidenced in 18 of the drawings, was houses. These houses tended to be small, closed, and drawn monochromatically and pre-schematically by mostly using shapes to depict the house. The houses were empty, drawn without details or a reference to the interior of the home, and lacked vitality as reflected in low embellishment and a small color palette, indicating low emotional investment. In addition, there were no figures or surroundings or any details. Even though they still lived at home with their spouses, in some of the drawings, the woman and her children were drawn at some distance from the house possibly reflecting a feeling of inferiority and a sense of emotional exclusion from the homes of their husbands. When figures were drawn, they were tiny, body-less, primitive, and anonymous (see Figures 3, 4). Obviously, it is possible, that some of the women may have found it difficult artistically to drawing some of the details of the house but the general feeling elicited by the drawings was a sense of marginalization, alienation, loneliness, and abandonment. In 12 of the narratives, there was a sense of death and no hope. This was echoed in the narratives: “there is no life in our home, no feeling of belonging, it feels like the house is not ours, it has no meaning either to the children or to me”; “I was very young when I married. My parents forced me to marry. It was an arranged marriage. I’m discouraged by the situation”; “I have no hope”; “I only stay with him because of the children.”

### Third Phenomenon: The Absentee Father and the Estranged Mother

**FIGURE 5:**
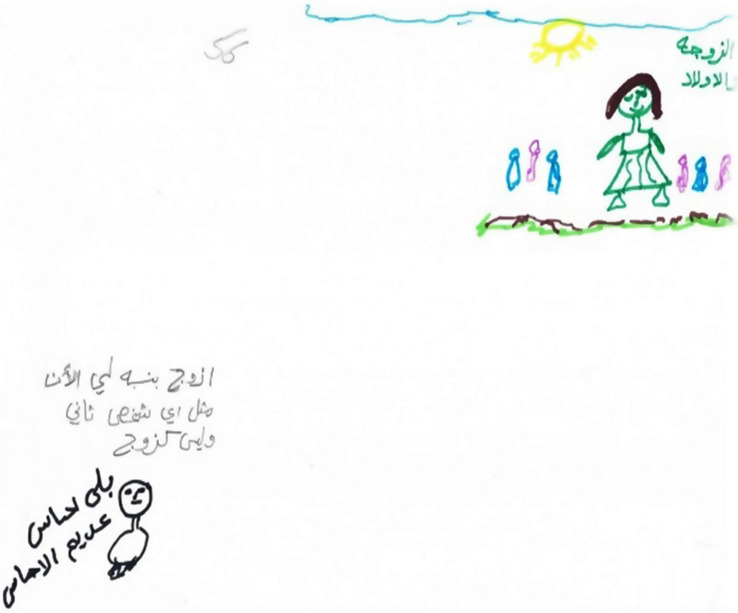
An example of “the Absentee Father or Mother” drawing.

**FIGURE 6:**
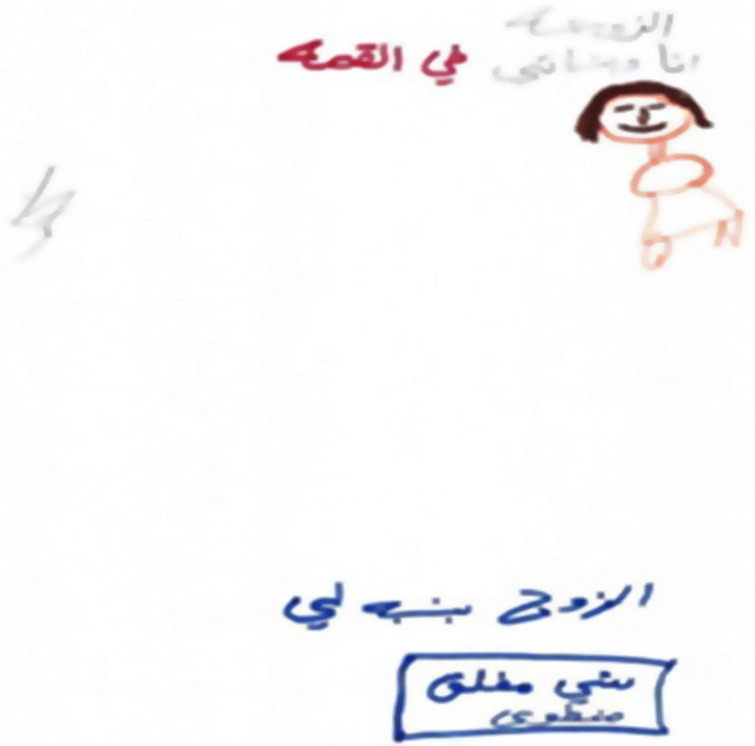
An example of “the Absentee Father or Mother” drawing.

The third phenomenon, which was identified in 27 drawings, was labeled the absentee father and the estranged mother. These drawings showed a family in which the father or the mother was absent or were drawn at a distance from the family, creating a sense of “presence-absence,” isolation and ostracism. This absence or separateness was represented by the use of different/opposite colors or by drawing the figure differently from the other figures/objects on the page and placing the spouses’ figures at different edges of the page. In many of the drawings, the women left a blank space between the mother and the father figures. In most cases, if the mother was drawn, her figure was tiny and missing body parts, such as her mouth or hands, creating a sense of powerlessness and silencing (see Figures 5, 6).

The women’s narratives reflected the sense that they had given up on their husbands, felt jealous of the other wife (wives), and even harbored revenge fantasies. To deal with the situation, in the drawings they appear to have nullified the husband, perhaps as a tactic to feel less trapped in the marriage. Some of the comments in the narratives included: “I only want to be strong for the children. I am indifferent to my husband; he is just a man; I do not treat him like a husband.” “My husband made a mistake when he married another woman; unfortunately, he is thinking of marrying another woman again, I am disappointed in him, I do not need him, the main thing is that I am with the children and they are fine and healthy. For me, he does not exist, I exist only for the children,” “My dream is to get rid of the first wife and to establish a good, warm relationship with my husband. I want him only for myself, but you can’t change reality. It’s just a dream.”

### Fourth Phenomenon: Drawings Incorporating Graphic Symbols

**FIGURE 7:**
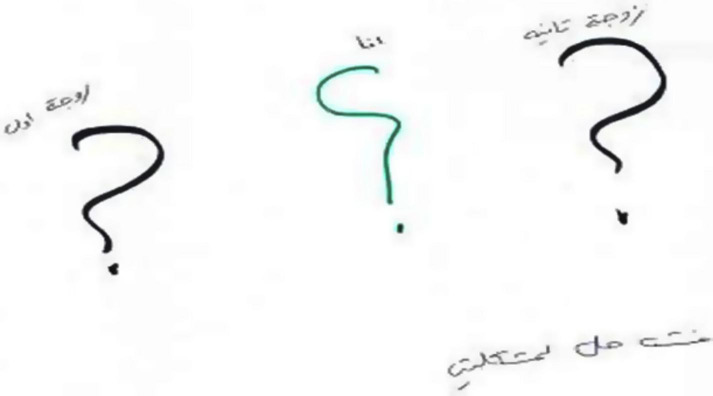
An example of a drawing incorporating written/graphic symbols.

**FIGURE 8:**
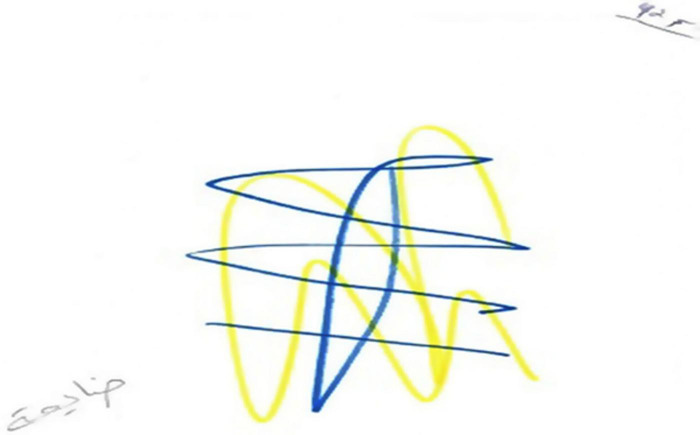
An example of a drawing incorporating written/graphic symbols.

The fourth phenomenon was characterized by drawings incorporating symbols and letters expressing distress (*n* = 13). The drawings in this category included scribbles, icons, and universal symbols as such as arrows to denote directions, mathematical symbols (=, +, ≠, question marks, × marks, and labyrinths. The drawings evoked feelings of confusion, sadness, anger, and astonishment as though the women were attempting to make sense of the situation and wanted to make sure that others could understand their experience in particular by asking “how can this be?” (see Figures 7, 8). This impression of confusion, perplexity, and injustice was also manifested in women’s narratives: “we walk in a big maze; nobody knows where he is and what he wants. Everything is messy and dark, painful and confusing, fear, despair, and helplessness are the family situation”; “a storm of emotions is in my heart, I do not understand why this has happened, what I did wrong to him, confused, do not understand everything, full of problems, quarrels, and shouting at home. Life went wrong for me.”; “full of unanswered questions, I’m in the middle between my husband and his second wife, wondering what I am for/to him.”

### Fifth Phenomenon: Growth and Development

**FIGURE 9:**
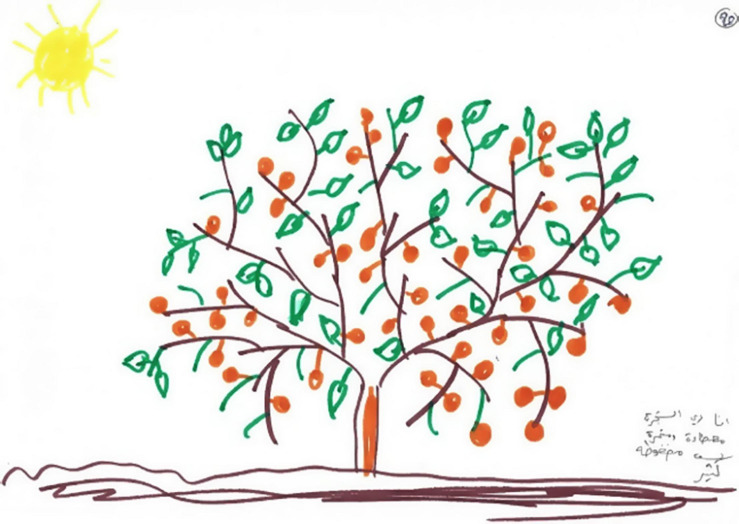
An example of a growth/development drawing.

**FIGURE 10:**
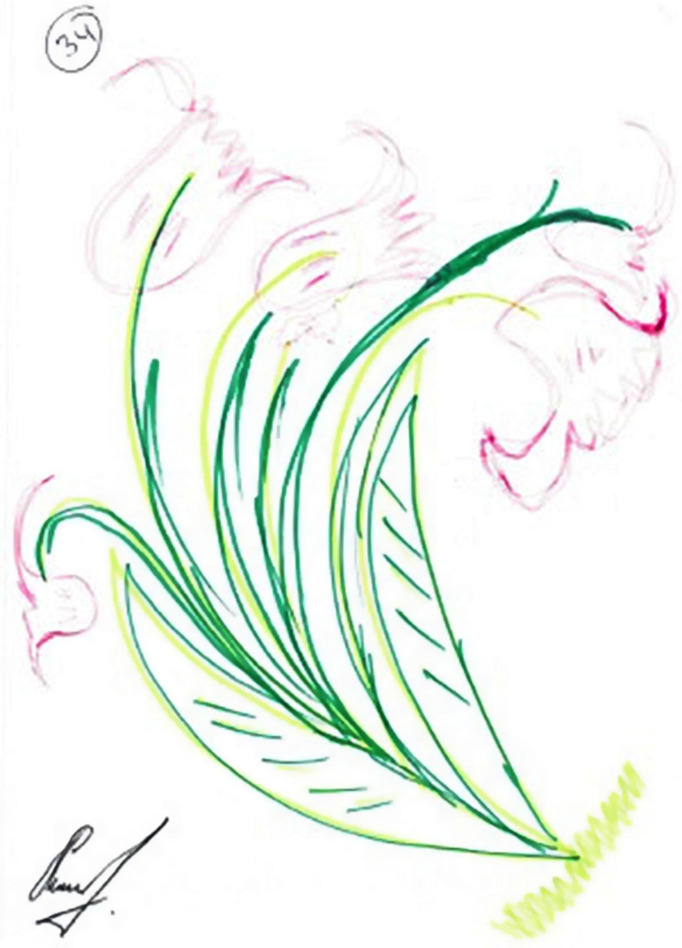
An example of a growth/development drawing.

The fifth phenomenon was termed growth and development. This was manifested in eight drawings and was reflected in images of blooming trees, birds, and flowers. By contrast to the paintings classified as pseudo-sweetness, the graphic style of the drawings was characterized by emotional investment and vitality as manifested in richness, colorfulness, decorativeness, and movement (see Figures 9, 10). All of these women’s narratives expressed hope and the desire for development, growth, and recovery. In the narratives, these women discussed their strengths and dealt with their and their children’s future with or without their husbands, saying “I am like a tree, fertile, giving, and generous, but I am under stress. I want to continue my life and support my children; they give strength to my life now. I want to see them successful”; “I am happy with my life, but if my husband is thinking of marrying a third wife, I will divorce him and continue living with or without him”; “I will move forward.”

## Discussion

The current study employed a projective art-based technique along with short verbal narratives to explore the experience of women living in polygamous families, who had experienced a traumatic marital relationship and were being treated by Israeli health and welfare services. Although most of the drawings communicated the experience realistically through the use of houses and human figures and attempted to depict a normative life, their drawings also revealed negative feelings of loneliness, helplessness, confusion, and internal distress. In addition, the longing for romantic relationships, jealousy, anger, and the desire for revenge and justice emerged from the narratives. These findings lend weight to previous studies suggesting that drawings enable painful experiences of disclosure by bypassing dissociative mechanisms (Amir and Lev-Wiesel, 2007). The findings are also in line with studies of women living in polygamous families, which have pointed to the range of mental problems these women face (Al-Krenawi, 2001, 2012; Al-Krenawi and Graham, 2006; Al-Krenawi and Slonim-Nevo, 2008b; Daoud et al., 2014).

However, about a quarter of the women employed dissociative drawing techniques such as adding cheerful/sweet objects such as flowers, hearts, or butterflies to circumvent the distressing experience (Pianta and Longmaid, 1999; Goldner and Scharf, 2012). The classical approach in the dissociation literature views dissociation as a pathological response to trauma in which the victims splits daily reality into the part that functions appropriately and the abusive, intolerant experience (Nijenhuis et al., 2010; Lahav and Elklit, 2016). However, using dissociative mechanisms in drawings and narratives may also be considered a normative survival technique since detachment from painful experiences minimizes stress and may protect these women from their overwhelming pain (Spiegel et al., 2011; Stein et al., 2013).

In the drawings, both the home and the family dynamics dominated. According to projective theories, house drawings can shed light on the individual’s sense of security, protection and support, and the interaction between their inner and outer worlds (Lev-Wiesel and Kleinberg, 2002; Huss, 2007; Yu et al., 2016; Sheng et al., 2019). Similarly, a family drawing can shed light on family dynamics such as physical intimacy or distance, the emotional tone in the home setting, aspects of closeness, isolation, rejection, and dominance among different family members (Lev-Wiesel and Samson, 2001; Lev-Wiesel and Kleinberg, 2002).

In the current study, the women’s houses were empty, lacked details and vitality. They were drawn in a monochromatic and careless way that instilled a sense of loneliness, ostracism, and abandonment. Almost a fifth of the drawings lacked a father figure. In others, the women were isolated or apparently rejected from their families. The absence of a father figure was previously found in drawings of children in polygamous families (Lev-Wiesel and Al-Krenawi, 2000). A sense of separation, isolation, or estrangement have been reported in drawings of mothers who are victims of intimate partner violence (Backos and Samuelson, 2017). The separation between the mother and child figures have been found in family drawings of mothers of children with Down syndrome who tend to draw a larger number of barriers between themselves and their children (Lev-Wiesel and Zeevi, 2007). The house/home, which is supposed to provide these women with a sense of security and protection, was depicted as a place promoting isolation, which may have represented difficulties in the family relationship (Elbedour et al., 2006). At other times it could have indicated the women’s longing for an intimate connection or their wish to punish their husband by distancing the father figure from their family.

In a large proportion of the drawings, a large space was left blank, and the drawings only depicted tiny anonymous figures who lacked body parts such as hands and eyes and were drawn in a restricted, careless, and limited way. The drawings of tiny and anonymous figures is thought to indicate vulnerability, incompetence, powerlessness, and non-existence (Nagamey et al., 2018), whereas a careless drawing indicates feelings of tension and anger toward the figure and is characteristic of an avoidant attachment style (Fury et al., 1997). For example, in a study conducted by Lev-Wiesel and Kleinberg (2002) who treated a battered woman, all the participants drew themselves smaller in size than the husband figure. In one-fifth of the drawings, the figure of the wife was lower on the page in relation to the figure of the husband.

Fragmented, detached, or omitted hands may reflect helplessness and incompetence, and the omission of the mouth may express the women’s inability to make their voice heard (Lev-Wiesel, 2005). In light of the literature on polygamy (Al-Krenawi, 2012, 2013), it comes as no surprise that these were the participants’ feelings of helplessness and despair. To overcome feelings they cannot support, the women described their tendency toward parentification in their narratives (Minuchin, 1974; Earley and Cushway, 2002); i.e., when parents turn to their children for emotional support and comfort. This kind of relationship, which may derive from situations such as parental conflict or divorce, households with domestic violence, and maternal helplessness (Fitzgerald et al., 2008; Bifulco et al., 2014), can become destructive when characterized by exaggerated emotional and instrumental responsibility (Byng-Hall, 2008).

Several drawings in the study incorporated written symbols and lettering that expressed attempts to communicate what these women perceived as an impossible situation. Using familiar symbols and icons, they maximized the possibility of being understood and evidenced their attempts to make sense of the situation that had been forced upon them (Huss et al., 2012). In these drawings, black dominated. In some cases, black was combined with red. Color choice is assumed to represent one’s emotional state (Lev-Wiesel and Daphna-Tekoha, 2000). Studies have indicated that while emotionally well-adjusted individuals respond to color openly, emotionally distressed people shy away from colors, and use limited color scales (Gantt and Tabone, 1998). There is a broad consensus across cultures that black represents death, mourning, or mortification, whereas red signifies love, passion, energy, power, fertility, blood, pain, anger, struggle, and revenge (Eisenbach et al., 2015). This combination may reflect the psyche’s natural inclination to move from destruction to life and achieve a sense of control (Lev-Wiesel, 2005). Empirically, several studies involving participants who endured “life-threatened experiences” (including adults who were sexually abused as children, earthquake victims, and Palestinian adults who must go through searches at Israel Defense Forces checkpoints to reach their schools or places of employment) revealed a substantial prevalence for red and black in their artworks (Gregorian et al., 1996; Eisenbach et al., 2015; Nagamey et al., 2018). In the present study, black and red may have disclosed these women’s despair and agony, anger, and desire for retaliation and justice.

A similar constellation also emerged from some of the women’s narratives that pointed to their feelings of jealousy and a desire for revenge. Life in a state of constant marital conflict, isolation, and feelings of injustice in polygamous families may elicit jealousy between the women involved (Al-Krenawi, 2012). Nevertheless, although anger, resentment toward the husband, and jealousy of the other wives appeared in women’s narratives (Guerrero, 1998), most felt pushed into a corner, and were only left with a desire for revenge. Their drawings distanced the husband from their children and created a separate family unit composed of themselves and their children. This kind of fantasy may help these women retain a certain degree of self-worth, reinstate justice, and restore emotional equanimity (Orth, 2004; McClelland, 2010). In a sense, the desire for revenge constitutes a form of narcissistic restoration, enabling a redefinition of the self, encouraging progression and ego stability, and preserving the person’s belief in a just and ordered society (McClelland, 2010; van Denderen et al., 2014).

Finally, by using images from nature and especially green flourishing trees, several women expressed the aspiration to live their life beyond surviving while fulfilling their potential and achieving positive self-regard and purpose in life. Researchers have suggested that drawings of a tree express one’s self-image and emotional state, and represent individuals’ urge to grow and prosper (Hammer, 1980; Stanzani Maserati et al., 2015). While bare, dehydrated, and rootless trees reflect feelings of powerlessness and helplessness (Mizuta et al., 2002), a healthy growing tree mirrors a person’s positive development (Isaksson et al., 2009). In the current study, the women’s trees may represent the concept of “thriving” (Diener et al., 2010; Su et al., 2014), which refers to higher-level functioning that reaches beyond the restoration of homeostasis after an adverse event (Ryff and Singer, 2003).

## Limitations and Conclusion

Several limitations of this study should be acknowledged. First, the sample was composed exclusively of Israeli Muslim Arab women who were in contact with the social services. Future studies should be conducted in polygamous families from other cultures and among well-functioning families to enable generalization. This study was based on women’s drawings and short narratives. Using additional research methods such as in-depth interviews or self-report questionnaires could extend the conclusions. Nevertheless, despite these limitations, the results shed light on the disturbing reality of isolation and despair of Arab women in polygamous families in the current sample. They also revealed the mechanisms that women employ to bypass their distress, such as parentification, creating a separate family unit, creating revenge fantasies, and seeking justice and dissociation. Nevertheless, some women exhibited strengths and expressed hopes of thriving.

## Data Availability Statement

The datasets presented in this article are not readily available because ethical consideration. Requests to access the datasets should be directed to the second author.

## Ethics Statement

The studies involving human participants were reviewed and approved by the Faculty of Social Welfare and Health Sciences, University of Haifa Ethics Committee (approval # 1809/19). The patients/participants agreed to participate in this study.

## Author Contributions

FG conducted the drawing tasks and narrative collection. Both authors analyzed the drawings and narratives and were involved in writing the article.

## Conflict of Interest

The authors declare that the research was conducted in the absence of any commercial or financial relationships that could be construed as a potential conflict of interest.
